# Dengue virus infection induces interferon-lambda1 to facilitate cell migration

**DOI:** 10.1038/srep24530

**Published:** 2016-07-26

**Authors:** Yu-Lin Hsu, Mei-Yi Wang, Ling-Jun Ho, Jenn-Haung Lai

**Affiliations:** 1 Institute of Preventive Medicine, National Defense Medical Center, Taipei, Taiwan, R.O.C; 2 Division of Allergy, Immunology, and Rheumatology, Department of Internal Medicine, Chang Gung Memorial Hospital, Chang Gung University, Tao-Yuan, Taiwan, R.O.C; 3 Institute of Cellular and System Medicine, National Health Research Institute, Zhunan, Taiwan, R.O.C; 4 Graduate Institute of Clinical Research, National Defense Medical Center, Taipei, Taiwan, R.O.C

## Abstract

A marked increase in the rate of dengue virus (DENV) infection has resulted in more than 212 deaths in Taiwan since the beginning of 2015, mostly from fatal outcomes such as dengue hemorrhagic fever and dengue shock syndrome. The pathogenic mechanisms of these fatal manifestations are poorly understood. Cytokines induce an overwhelming immune reaction and thus have crucial roles. Interferon-lambda (IFN-λ), a newly identified IFN subtype, has antiviral effects, but its immunologic effects in DENV infection have not been investigated. In the present study, we show that DENV infection preferentially induced production of IFN-λ1 in human dendritic cells (DCs) and human lung epithelial cells. Virus nonstructural 1 (NS1) glycoprotein was responsible for the effect. DENV-induced production of IFN-λ1 was dependent on signaling pathways involving toll-like receptor (TLR)-3, interferon regulation factor (IRF)-3, and nuclear factor-kappaB (NF-κB). Blocking interaction between IFN-λ1 and its receptor IFN-λR1 through siRNA interference reduced DENV-induced DC migration towards the chemoattractants CCL19 and CCL21, by inhibiting CCR7 expression. Furthermore, IFN-λ1 itself induced CCR7 expression and DC migration. Our study presents the first evidence of the mechanisms and effects of IFN-λ1 induction in DENV-infected DCs and highlights the role of this cytokine in the immunopathogenesis of DENV infection.

Dengue virus (DENV) is a positive-strand RNA virus belonging to *Flaviviridae* family of viruses. DENV infection has long been a major public health concern worldwide, particularly so in Asian countries^1^. According to a report from the Centers for Disease Control of Taiwan (R.O.C.), more than 43,060 people were infected by DENV, among whom more than 212 died, since the beginning of 2015. The causes of death in DENV-infected patients are most likely dengue hemorrhagic fever (DHF) and dengue shock syndrome (DSS). However, the pathogenic mechanisms that lead to these severe clinical manifestations are not clear. The correlation between viral load and disease severity is not strong; thus, the host reaction to virus infection, which results in the release of high levels of cytokines and other soluble mediators, is believed to have an important role in these fatal sequelae^2^,^3^.

The interferons (IFNs) are an important group of cytokines that are induced during viral infection and have strong antiviral and immunologic activities^4^,^5^. There are three IFN subtypes: the type I IFNs (IFN-α/β), type II IFN (IFN-γ) and type III IFNs (IFN-λ). The type III IFNs comprise four members—IFN-λ1, IFN-λ2, IFN-λ3 and IFN-λ4—which arise from gene duplication^6^,^7^. IFN-λ binds to a receptor complex containing two subunits, IFN-λ receptor 1 (IFN-λR1) and interleukin (IL)-10R2, and, like type I IFNs, mediates antiviral activity through the Janus kinase (JAK)-signal transducer and activator of transcription (STAT) pathway and induction of IFN-stimulated genes (*ISGs*). However, the IFN-λ–mediated effects are independent of the type I IFNs^6^,^8^.

Although IFN-α/β and IFN-λ have certain similarities in intracellular signaling pathways, IFN-λ preserves several biological characters that are distinct from those of IFN-α/β. IFN-λ and IFN-α/β bind different membrane receptors, to transduce signals^9^. The effects of IFN-λ are prominent in epithelial cells that are constantly exposed to commensal and pathogenic microbes^10^,^11^. In a murine model of norovirus infection, the IFN-λ but not the IFN-α/β pathway is crucial in antibiotic-mediated prevention of persistent enteric norovirus infection^12^. Noticeably, there is evidence showing that the peroxisomes were the primary site that initiates type III IFN expression, complementary with the actions of the type I IFN responses induced from mitochondria^13^. Furthermore, genome-wide association studies (GWASs) identified that both *IFN-λ3* and *IFN-λ4* genes were linked to clearance of viruses such as hepatitis C virus, human cytomegalovirus and herpes simplex virus 1^7^,^14^,^15^. In clinical applications, the focused nature of IFN-λ–mediated signaling pathways suggests that IFN-λ is less likely to result in the adverse events associated with the clinical use of IFN-α/β^14^,^16^.

We previously demonstrated that human dendritic cells (DCs), the most efficient antigen-presenting cells, can be infected by DENV^17^, and several IFN signaling–related genes are induced in DENV-infected DCs^18^. In addition to DCs, the human lung epithelial cell line A549 has long been adopted for studying the effects and mechanisms of DENV infection, especially in investigations of the effects of IFNs^19^,^20^. Because the role of IFN-λ in DENV infection remains largely unknown, we investigated how DENV regulates IFN-λ production and the effects of IFN-λ in human DCs.

The results demonstrate that DENV infection preferentially induced production of IFN-λ1 in DCs and the human lung epithelial cell line A549 via its nonstructural 1 (NS1) glycoprotein. Induced IFN-λ1 mediated DC migration and blocking interaction between IFN-λ1 and its receptor IFN-λR1 reduced DENV-induced DC migration. The present study also examined the signaling events involved in DENV-induced IFN-λ1 production. The results in this report suggest that adequate control of IFN-mediated immunologic effects may need to include IFN-λ1 blocking.

## Results

### DENV infection induced IFN-λ production in DCs

**Figure 1:**
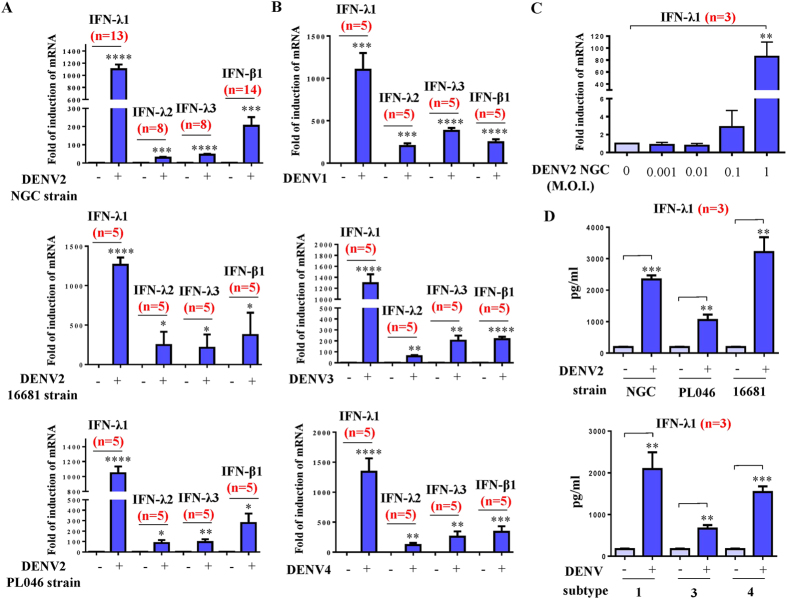
Dengue virus (DENV) infection induced IFN production in human DCs. Human DCs (1 × 10^6^ cells/ml) were infected by mock or different strains of DENV2, including NGC, 16681 and PL046 strains at a MOI of 5. Quantitative RT/PCR was used to determine expression of mRNAs of the *IFN-λ1*, *-λ2*, *-λ3* and *-β1* genes (**A**). Similarly, mRNA levels of these IFNs were determined in DCs infected by different subtypes of DENV (**B**). The mRNA levels of IFN-λ1 in DCs infected by the DENV2 NGC strain at different MOIs were measured (**C**). The protein levels of IFN-λ1 in supernatants collected from different conditions were determined by ELISA (**D**). The data shown are from various numbers of different donor DCs as indicated (n). The analysis was performed by ANOVA or Student’s t test. *p < 0.05; **p < 0.01; ***p < 0.001, ****p < 0.0001.

DCs were infected by mock or different strains of DENV2, including the NGC, 16681 and PL046 strains (A), or DENV1, DENV3 or DENV4 (B), at a multiplicity of infection (MOI) of 5, after which the cells were collected to measure mRNA expression of IFNs. DENV infection induced expression of IFN-λ1, IFN-λ2, IFN-λ3 and IFN-β1 mRNA in DCs (Fig. 1A,B). Among these IFNs, IFN-λ1 was highly induced. Induction of IFN-λ1 could be detected at a low MOI (0.1) of DENV2 infection (Fig. 1C). Induction of IFN-λ1 in DENV-infected DCs was also demonstrated in analysis of protein levels (Fig. 1D).

### DENV-induced IFN-λ1 production was dependent on Toll-like receptor (TLR)-3

**Figure 2:**
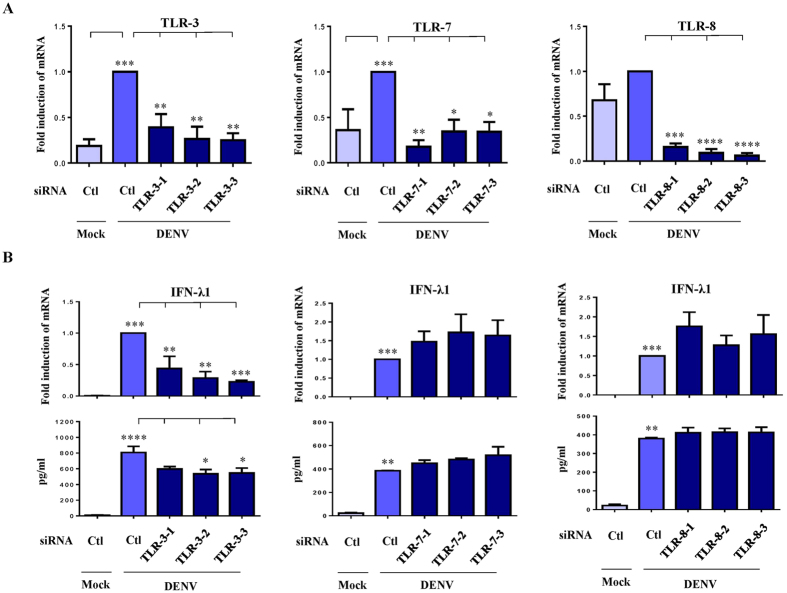
DENV-induced IFN-λ1 expression was dependent on TLR-3 in DCs. Human DCs were transfected with control siRNA (siCtl) or three sets of duplexes of TLR-3, -7 or -8 siRNA for 24 h and then infected by mock or DENV for an additional 48 h. Cells were collected and mRNA levels of individual TLRs were determined (**A**). In parallel, the levels of IFN-λ1 mRNA and protein were determined by quantitative RT/PCR and ELISA, respectively (**B**). The data show results pooled from at least three independent experiments. The analysis was performed by ANOVA, as described in the Methods. *p < 0.05, **p < 0.01, ***p < 0.001, ****p < 0.0001.

Toll-like receptors are involved in various forms of stimuli-induced IFN production^21^. We used gene knockdown with small interfering RNAs (siRNAs) to determine the roles of TLRs in DENV-induced IFN-λ1 production. Detection of mRNA levels show that TLR-3, -7 and -8 were successfully knocked down (Fig. 2A). A deficiency of TLR-3 but not of TLR-7 or -8 reduced IFN-λ1 mRNA expression and protein production in DENV-infected DCs (Fig. 2B). Knockdown of TLR-3 but not of TLR-7 or -8 also inhibited DENV-induced IFN-λ2, -λ3 and -β1 mRNA expression and protein production (Supplementary Figure 1).

### Virus NS1 glycoprotein was responsible for IFN-λ1 production

**Figure 3:**
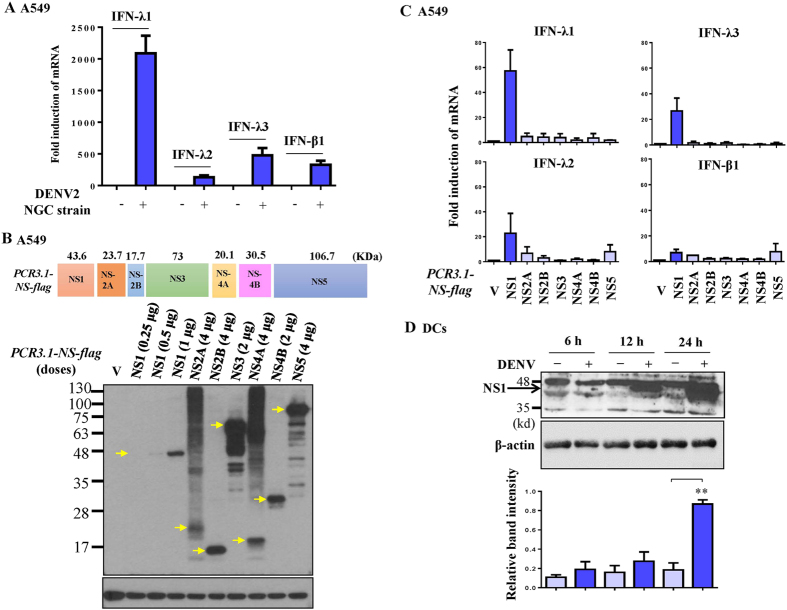
DENV-induced IFN-λ1 expression was through NS1 in human lung epithelial cell line A549. A549 cells were infected by DENV, and the mRNA levels of IFNs were determined (**A**). Transfection of various doses of plasmids encoding different components of viral NS genes (*PCR3.1-NS-flag*) into A549 cells (1 × 10^5 ^cells/ml) resulted in varying expression of viral NS proteins, as determined by Western blotting using equal amount of loading protein for analysis (**B**). Various amounts of plasmids containing viral NS genes corresponding to the equivalent viral NS proteins measured by Western blotting were transfected into A549 cells and the fold induction of mRNAs of IFNs was determined by RT/PCR (**C**). The expression of NS1 in human DCs infected by DENV for various time points was determined by Western blotting (**D**). The data show representative results and analysis pooled from three independent experiments examining different donor DCs. **p < 0.01.

To identify the viral component responsible for production of IFN-λ1, the human lung epithelial cell line A549 was chosen because of its accessibility for transfection and gene expression in our previous system^18^. We reproducibly detected induction of IFN-λ1 mRNA in A549 cells after DENV infection (Fig. 3A). Figure 3B show that, although expression levels varied, virus NS proteins were successfully induced in A549 cells after transfection with expression plasmids encoding different viral *NS* genes. Various amounts of plasmids containing viral *NS* genes corresponding to the equivalent viral NS proteins measured by Western blotting were transfected into A549 cells and the fold induction of mRNAs of IFNs was determined. The results show that NS1 glycoprotein was the main viral component responsible for DENV-induced IFN-λ1 mRNA expression (Fig. 3C). The expression of NS1 could be readily detected in DCs infected by DENV (Fig. 3D).

### NS1-induced IFN-λ1 production was dependent on NF-κB activation

**Figure 4:**
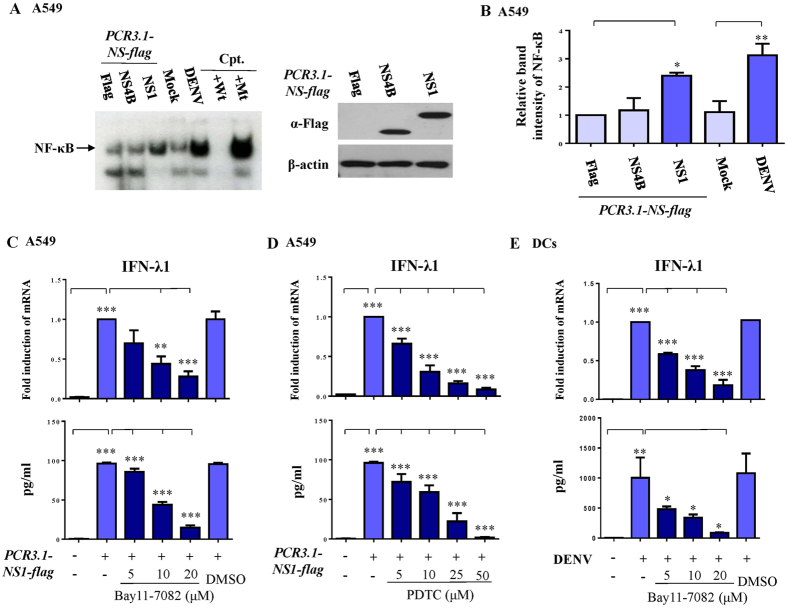
DENV NS1 glycoprotein induced IFN-λ1 production through activation of NF-κB signaling. A549 cells infected by mock or DENV or transfected with a plasmid encoding NS1 or NS4B gene, as indicated, were collected. NF-κB DNA-binding activity was determined in nuclear extracts, as described in Methods (**A**). Nuclear extracts pre-incubated with wild-type or mutant NF-κB oligonucleotides served as controls. (**B**) shows analysis from at least three independent experiments. After transfection of a plasmid encoding NS1, A549 cells were treated with different doses of the NF-κB inhibitor Bay11-7082 (**C**) or PDTC (**D**), and the expression of IFN-λ1 mRNA and IFN-λ1 protein was determined by RT/PCR and ELISA, respectively. An approach similar to (**C**) was conducted, but DCs were studied instead (**E**). Cpt, competitors; Wt, wild-type; Mt, mutant. The analysis was performed by ANOVA. *p < 0.05, **p < 0.01, ***p < 0.001. Ctl, control.

We previously demonstrated that DENV infection induced several key signaling pathways in immune system activation^18^,^22^. Electrophoretic mobility shift assay (EMSA) analysis show that virus NS1 but not NS4B glycoprotein induced NF-κB DNA-binding activity in A549 cells (Fig. 4A,B). NS1-induced IFN-λ1 production in A549 cells was inhibited in a dose-dependent manner by NF-κB inhibitors such as Bay11-7082 (Fig. 4C) and pyrrolidine dithiocarbamate (PDTC) (Fig. 4D). As predicted, knockdown of TLR-3 did not affect NS-1-induced IFN-λ1 mRNA expression in A549 cells (data not shown). Furthermore, DENV-induced IFN-λ1 production in DCs was susceptible to inhibition by the NF-κB inhibitor Bay11-7082 (Fig. 4E).

### Induction of IFN-λ1 was interferon regulation factor (IRF)-3–dependent

**Figure 5:**
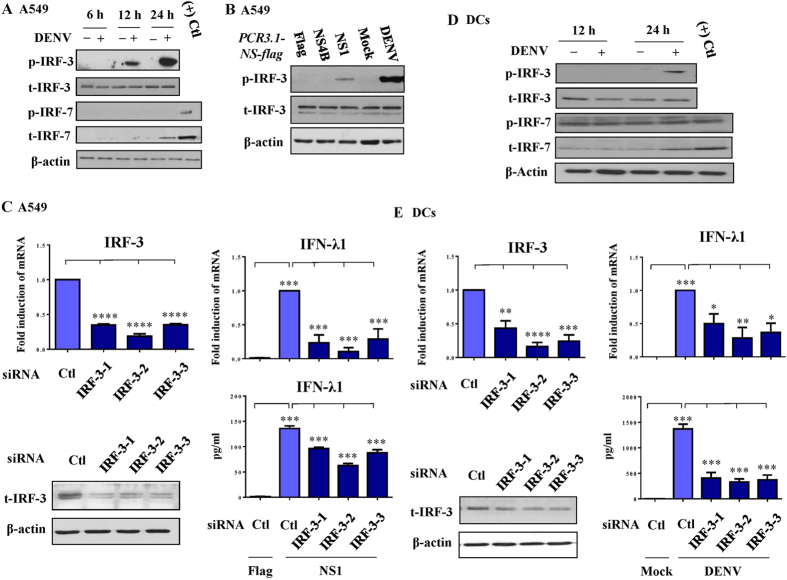
DENV-induced and NS1-induced IFN-λ1 expression was IRF-3-dependent. A549 cells were infected by mock or DENV for different time periods, and the expression of total or phosphorylated IRF-3 and -7 was determined by Western blotting (**A**). Expression of total or phosphorylated IRF-3 in A549 cells transfected with plasmids encoding NS1 or NS4B was determined (**B**). The siRNA interference approach with three sets of duplexes successfully knocked down expression of IRF-3, which reduced NS1-induced IFN-λ1 mRNA expression and protein production (**C**). Similar to the experiments shown in (**A**,**C**), DENV-induced IRF-3 activation and the effect of IRF-3 knockdown were determined in human DCs (**D**,**E**, respectively). The data show representative results and analysis pooled from at least three independent experiments examining different donor DCs. The analysis was performed by ANOVA, as described in the Methods. *p < 0.05, **p < 0.01, ***p < 0.001, ****p < 0.0001.

Analysis of the potential involvement of IRF-3 and -7 in DENV-induced IFN-λ1 expression show that DENV infection for 12–24 h increased levels of phosphorylated IRF-3 but not IRF-7 in A549 cells (Fig. 5A). Transfection and expression of NS1 but not of NS4B increased phosphorylated IRF-3 levels in A549 cells (Fig. 5B). To determine the roles of IRF-3 in DENV-induced IFN-λ1 expression, three different duplexes of IRF-3 siRNA were used to knockdown IRF-3, as described in the Methods section. As shown in Fig. 5C, three different siRNAs of IRF-3 successfully suppressed IRF-3 mRNA and protein levels in A549 cells. Knockdown of IRF-3 suppressed DENV-induced (data not shown) and DENV NS-1-induced IFN-λ1 mRNA expression and protein levels in A549 cells (Fig. 5C). DENV-induced activation of IRF-3 was also observed in DCs (Fig. 5D) and, similarly, knockdown of IRF-3 reduced DENV-induced IFN-λ1 mRNA expression and protein production in DCs (Fig. 5E). DENV infection increased expression of total IRF-7 in A549 cells and DCs (Fig. 5A,D); however, the mechanisms underlying these effects are unknown. Because IRF-1 was implicated playing a role in DENV-induced IFN-λ1 production in Huh7 cells^13^, in supportive, our results revealed the enhancement of nuclear translocation of IRF-1 from cytosol after DENV infection in both A549 cells and DCs (Supplementary Figure 2).

### Knockdown of IFN-λR1 impaired DENV-induced DC migration

**Figure 6:**
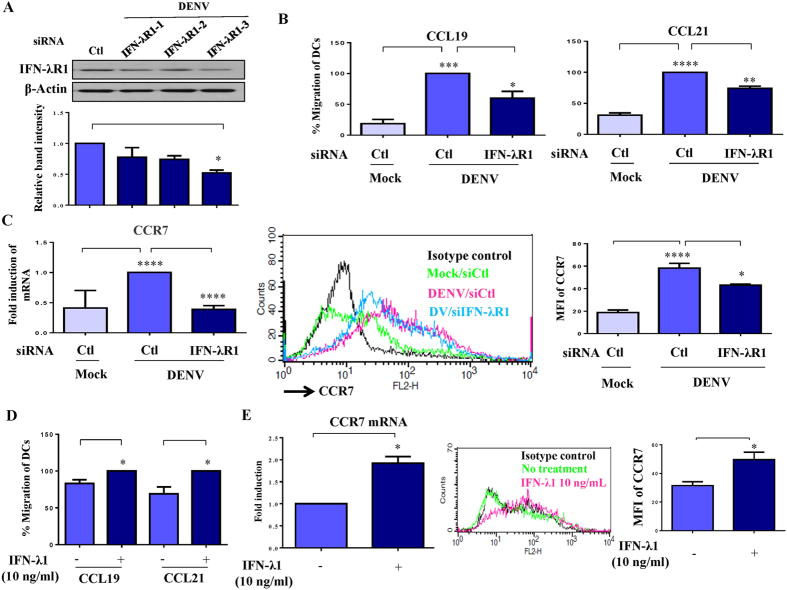
Defective migration of DENV-infected DCs with reduction of IFN-λR1. Human DCs were transfected with control siRNA (siCtl) or three sets of duplexes of IFN-λR1 siRNA for 24 h and then infected with DENV for an additional 48 h. Cells were collected, and the levels of IFN-λR1 protein (**A**) were determined. In parallel, cells were collected for measurement of chemotactic activity by transwell assays, using CCL19 or CCL21 as a chemoattractant (**B**). Human DCs transfected with control siRNA (siCtl) or IFN-λR1 siRNA (siIFN-λR1) for 24 h were infected by mock or DENV for an additional 48 h. Cells were collected for measurement of CCR7 mRNA levels by quantitative RT/PCR and cell surface expression by flow cytometry (**C**). The effects of recombinant IFN-λ1 on DC migration (**D**) and CCR7 mRNA and cell surface expression (**E**) were determined. MFI, mean fluorescence intensity. The data show results pooled from at least three independent experiments examining different donor DCs. The analysis was performed by ANOVA. *p < 0.05, **p < 0.01, ***p < 0.001, ****p < 0.0001. Ctl, control.

To determine the effect of IFN-λ1 on DENV infection, we used a genetic interference approach to reduce the expression level of IFN-λR1. Although three IFN-λR1 duplexes successfully knocked down expression of IFN-λR1 mRNA (Supplementary Figure 3A), only one duplex (IFN-λR1-3) significantly reduced IFN-λR1 protein level, as determined by Western blotting (Fig. 6A). Consistent with the antiviral activity of IFN-λ1, IFN-λR1 knockdown increased viral mRNA level (Supplementary Figure 3B); however, IFN-λR1 knockdown did not affect expression of maturation or activation markers on DENV-infected DCs (Supplementary Figure 3C,D). After pathogen infection in the periphery, DCs mature and migrate from periphery to lymph nodes^22^,^23^. Chemotaxis assays were used to determine whether knockdown of IFN-λR1 affects DENV-induced migration of DCs. The results indicate that DENV infection induced DC migration toward both CCL19 and CCL21 chemoattractants. The effects were inhibited in DENV-infected DCs deficient in IFN-λR1 (Fig. 6B). In support of the results of chemotaxis assays, IFN-λR1 knockdown suppressed both mRNA and cellular surface expression of CCR7, the receptor for CCL19 and CCL21 (Fig. 6C). The results further show that IFN-λ1 was capable of inducing DC migration (Fig. 6D) and increasing CCR7 mRNA level and cell surface expression (Fig. 6E).

### Knockdown of IFN-λR1 inhibited DENV-induced NF-κB and AP-1 DNA binding activity

**Figure 7:**
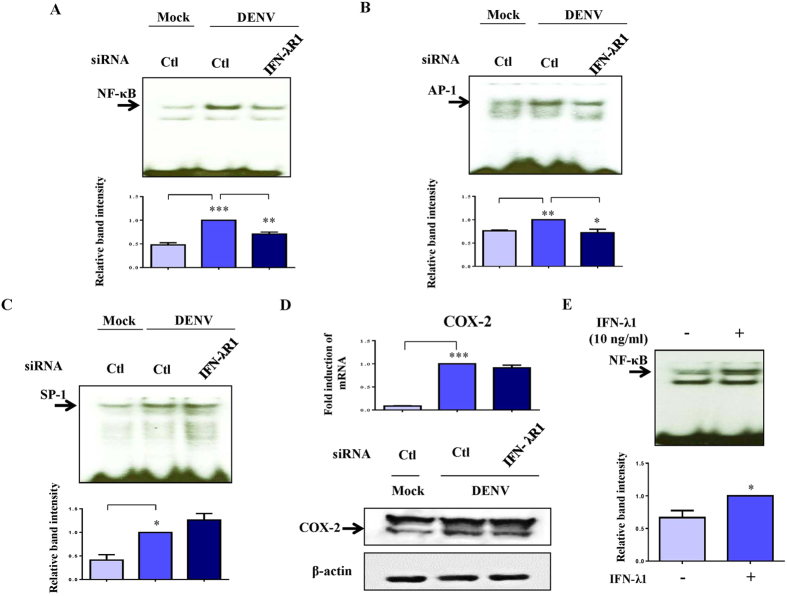
Decrease in DENV-induced activation of NF-κB and AP-1 in DCs with knockdown of IFN-λR1. Human DCs transfected with control siRNA or IFN-λR1 siRNA for 24 h were infected by mock or DENV for an additional 8 h. Cells were collected for measurement of NF-κB (**A**), AP-1 (**B**) or SP-1 (**C**) DNA-binding activity by EMSA or COX-2 expression by Western blotting (**D**). The NF-κB DNA-binding activity of DCs treated with or without IFN-λ1 was determined (**E**). The results pooled from at least three independent experiments using different donor cells are presented next to the representative figures. The analysis was performed by ANOVA. *p < 0.05, **p < 0.01, ***p < 0.001. Ctl, control.

**Figure 8:**
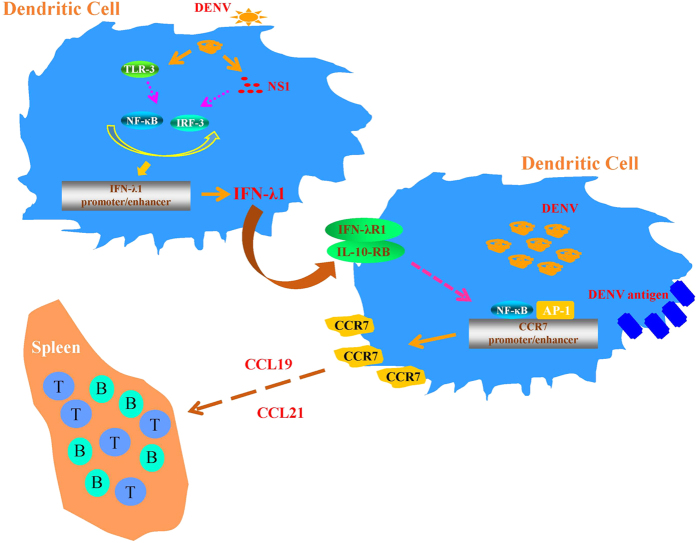
An illustration shows the effects of DENV-induced IFN-λ1 production in DCs. Infection of DCs by DENV induced expression and production of IFN-λ1, and this effect was dependent on TLR-3, NF-κB and IRF-3 signaling. Viral NS1 was the main viral component responsible for the observed effects on activating NF-κB and IRF-3 signaling. Through IFN-λR1 and IL-10-RB, IFN-λ1 activated the NF-κB and AP-1 signaling pathways, regulated CCR7 expression and facilitated cell migration from periphery to lymphoid organs like spleen.

DENV infection induced activation of several transcription factors that potentially regulate CCR7 expression. The mechanisms underlying this observation were investigated. As shown in Fig. 7A, NF-κB DNA-binding activity was greatly reduced in DENV-infected DCs with reduced IFN-λR1. In parallel, DENV-induced AP-1 DNA-binding activity was suppressed in cells with knockdown of IFN-λR1 (Fig. 7B); however, SP-1 DNA-binding activity was not affected (Fig. 7C). Although we previously observed that DENV infection activated cyclooxygenase-2 (COX-2) expression^22^, suppression of COX-2 expression did not occur under the condition of reduced IFN-λR1 (Fig. 7D). Consistent with induction of DC migration, IFN-λ1 treatment induced NF-κB DNA-binding activity (Fig. 7E). In order to further expand the knowledge of IFN-λR1 knockdown on production of other cytokines in DENV infection, the supernatants were collected for cytokine measurement. Interestingly, IFN-λR1 knockdown only affected DENV-induced IL-1β production. Several cytokines, including IL-6, -8, -10 and -12, and some chemokines, including macrophage inflammatory protein (MIP)-1α, MIP-1β, monocyte chemoattractant protein (MCP)-2 and regulated on activation, normal T cell expressed and secreted (RANTES) were not affected (Supplementary Figure 4). Figure 8 illustrates the mechanisms and effects of IFN-λ1 induction in DENV infection of the DC.

## Discussion

A recent study reported that serum levels of IFN-λ1 in DENV-infected patients were up to 1.8-fold those of healthy controls^24^. In addition, the authors found that IFN-λ1 was capable of inhibiting DENV2 replication in the epithelial cell line C33-A^24^. In the present report, we demonstrated that IFN-λ1 was induced in human DCs and the human lung epithelial cell line A549 after DENV infection. These results were further confirmed by examining different DENV subtypes and infection with different DENV2 strains. Virus NS1 glycoprotein appeared to be the principal viral component responsible for DENV-induced production of IFN-λ1. Several key signaling molecules, such as TLR-3, IRF-3, and NF-κB, were involved in DENV-induced IFN-λ1 production. The use of RNA interference to block interaction between IFN-λ1 and its receptor IFN-λR1 effectively reduced DENV-induced activation of NF-κB and AP-1, as well as CCR7 expression, which suppressed DC migration. Moreover, IFN-λ1 by itself induced CCR7 mRNA expression and caused DC migration. These results highlight the immunologic effects of IFN-λ1 in DENV infection.

The involvement of various TLR-mediated signaling pathways appears to be virus- and tissue-specific. Treatment of HepG2 cells with TLR-3 ligand but not with ligands for TLR-1/2, -2/6, -4, -5 or -7/8 effectively induced IFN-β to mediate antiviral effects^25^. In addition, TLR-3 was the only TLR that mediated IFN-λ production in airway epithelial cells^21^. TLR-9 stimulation by CpG DNA induced expression of three IFN subtypes in plasmacytoid DCs; however, only IFN-β and -λ were induced in monocyte-derived DCs—by lipopolysaccharide or poly I:C stimulation of TLR-4 or -3 signaling, respectively^26^. Through intravaginal administration of herpes simplex virus type 2 in mice, researchers demonstrated that TLR-9 stimulation induced a particularly strong IFN-λ response^27^. In the presence of virus infection, additional signals from TLR resulted in greater production of IFN-λ than that induced by virus infection alone^28^. Interestingly, DENV by itself was a weak stimulant that inhibited TLR-induced NF-κB activation and cytokine production^29^. The present results show that TLR-3 but not TLR-7 or -8 was involved in DENV-induced production of IFN-λ1 in human DCs. Knowledge of the differential participation of TLR-mediated signaling in virus infection may help in the development of therapeutic regimens against specific viral targets.

As compared with respiratory syncytial virus infection, human metapneumovirus (hMPV) infection induced a stronger IFN-λ response in A549 cells^30^. hMPV infection of A549 cells resulted in greater expression of IFN-λ2/3 than of IFN-λ1, among the IFN-λ members examined. Induction of IFN-λ2/3 expression by hMPV was dependent on IRF-7 but not on IRF-3^30^. Interestingly, both IRF-3 and -7 were essential factors in induction of the *IFN-λ*2 and *IFN-λ*3 genes; whereas induction of IFN-λ1 needed an additional activation signal from NF-κB in hepatitis C virus infection of hepatocytes^28^. Fibroblasts obtained from a patient with a hypomorphic mutation of the NF-κB essential modulator encoding the regulatory subunit of the IκB kinase complex, which results in defective activation of NF-κB and IRF-3, could not efficiently produce IFN-λ in response to TLR-3 stimulation^31^. According to Odendall *et al*.^13^ that knockdown of either IRF-3 or IRF-1 inhibited DENV-induced IFN-λ1 mRNA expression in Huh7 cells; however, compared to that from knockdown of IRF-1, the effect on knockdown of IRF-3 preserved more potent inhibitory effect. These authors further showed that sendai virus infection-induced phosphorylation of IRF-3 was not affected by IRF-1-knockdown suggesting that IRF-1 and IRF-3 are activated independently of each other^13^. Although DENV infection enhanced nuclear translocation of IRF-1 from the cytosol in both A549 cells and DCs (Supplementary Figure 2), the significance of this observation remains unclear. Our results provided solid evidence that at least IRF-3 played a pivotal role in DENV-induced IFN-λ1 production. It is likely that both IRF-1 and IRF-3 are important in DENV-induced IFN-λ1 production and there are possibly synergistic inhibitory effects by simultaneously blocking IRF-1 and IRF-3 on DENV-induced IFN-λ1 production.

NS1 glycoprotein and anti-NS1 antibodies are detectable in serum at the beginning of DENV infection, and NS1 levels positively correlate with disease severity^32^. Anti-NS1 antibodies recognize protein disulfide isomerase on platelets and inhibit platelet aggregation^33^. The NS1 glycoprotein interferes with the complement activation system, by binding to complement 4 (C4) and reducing C4b deposition and C3 convertase activity, resulting in evasion of immune protection^34^. Formation of NS1 and heterologous non-neutralizing antibody immune complex may also mediate complement activation and trigger plasma leakage^35^. Although coadministration of NS1 and a sublethal dose of DENV2 caused a lethal vascular leak syndrome^36^, immunization with a recombinant NS1 glycoprotein or a DNA plasmid encoding the NS1 gene was protective against DENV2 infection^36^,^37^,^38^. Recent reports demonstrated that purified NS1 induced production of proinflammatory cytokines and chemokines in human peripheral blood mononuclear cells, via differential usage of TLRs-mediated signaling pathways^39^,^40^. In the present report, we clearly detected the expression of NS1 within 12–24 h following DENV infection of DCs. Although the difference at 12 h time point of infection could not reach statistical significance, likely due to limited samples examined, the tendency of gradually increased expression of NS1 following DENV infection existed. It is also very likely that NS1 can, under the detectable protein level, initiate and activate its downstream signaling pathways and trigger responses. The activation of NF-κB DNA-binding activity, IRF-3 phosphorylation and IFN-λ1 production by NS1 but not by NS4B (as a control) clearly indicates its effects.

Migration of potent antigen-presenting DCs from periphery to lymphoid organs is a critical immunopathogenic step in DENV infection, as it leads to viral spread and initiation of the immune reaction, through priming of adaptive B- and T-cell responses^41^. IFN-λR1 deficiency impaired DENV-induced DC migration toward the chemoattractants CCL19 and CCL21, and reduction of the receptor of these two chemokines, CCR7, was responsible for the effect. Furthermore, our results show that IFN-λ1 by itself functioned as an autocrine that drove DC migration through induction of CCR7. The NF-κB- and AP-1-dependent signaling pathways but not the COX-2-dependent signaling pathway mediated these effects. In support of the present study, by chromatin immunoprecipitation assay and several different approaches, several functional NF-κB-binding sites were identified in the promoter/enhancer of *CCR7* gene^42^,^43^. Because DENV-induced COX-2 and galectin-9 expression also positively regulated DC migration as presented in our previous work^22^,^23^, as predicted, DENV infection-induced DC migration was stronger than that induced by IFN-λ1 treatment alone. A recent study showed that, in dogs with mammary cancer, myeloid-derived suppressor cells secreted IFN-λ, which promoted angiogenesis, epithelial–mesenchymal transition and invasion and migration of tumor cells^44^. Determining how IFN-λ1 induced in DENV infection regulated DC migration and by itself promoted such an effect will require future clinical studies of the roles of IFN-λ1 in persons infected with DENV. A recent report suggests that, among patients infected with DENV1 or 2, IFN-α levels were higher in those with dengue fever than in those with DHF^45^, which suggests that cytokines other than IFN-α are responsible for the overwhelming immune response in patients with DHF and DSS. In addition, serum IFN-β level poorly correlated with severity of dengue illness^46^. The study results in this report support a notion that IFN-λ1 is likely to be one of the primary mediators of the marked immune response seen in patients infected by DENV.

Given that IFN-λ1 could potentially be a major player among cytokines induced in DENV infection, the antiviral immunity of IFN-λ1 in the early phase of virus infection cannot be ignored. In response to host immunity, DENV also evolves many strategies such as inhibiting IFN production or targeting IFN signaling to attenuate the host’s innate immune responses^47^. While the STAT signaling pathways have long been recognized as the DENV targets to inhibit type I IFN signaling^48^, an adaptor protein named as stimulator of the interferon gene (STING), a molecule critical in inducing expression of type I IFN and exerting antiviral immunity^49^, appears to be another interesting target for DENV to antagonize the antiviral immunity of type I IFN^50^. The DENV NS2B3 protease complex has been shown to be responsible for degrading STING and inhibits type I IFN production in human cells^51^. Because there are certain similarities in signaling pathways regulating IFN-α and IFN-λ production, it is likely that DENV may also develop mechanisms to evade IFN-λ1-mediated antiviral immunity. Currently, we are exploring this possibility.

## Methods

### Culture medium and reagents

The culture medium consisted of RPMI 1640 and F12 (Gibco-BRL, Life Technologies Corporation, Carlsbad, CA, USA) supplemented with 10% fetal bovine serum (FBS, Hyclone, Thermo Fisher Scientific Inc, Waltham, MA, USA). Recombinant granulocyte-macrophage colony-stimulating factor (GM-CSF) and IL-4 were purchased from R&D (Minneapolis, MN, USA). The inhibitors for NF-κB activation, including Bay11-7082 and PDTC, were purchased from Calbiochem (Merck KGaA, Darmstadt, Germany) and Sigma (St. Louis, MO, USA), respectively. The fluorescent-conjugated antibodies (Abs) recognizing CD80, CD86, CD83 and HLA-DR were purchased from BD Pharmingen (BD Biosciences, CA, USA). Anti-CCR7, recombinant IFN-λ1 and chemoattractants, including CCL19 and CCL21, were purchased from R&D Systems. Anti-Flag antibody was purchased from Sigma. Anti-NS1 antibody was purchased from Genetax (GeneTex Inc, Irvine, CA, USA). Anti-phosphorylated IRF-3 and anti-phosphorylated IRF-7 were purchased from Cell Signaling (Beverly, MA, USA). Antibodies recognizing total IRF-1, total IRF-3, total IRF-7, IFN-λR1, COX-2 and upstream transcription factor2 (USF2) were purchased from Santa Cruz Biotechnology (Santa Cruz, CA, USA).

### Preparation of DCs and human lung epithelial cell line A549

DCs were prepared from purified CD14^+^ monocytes, as previously described^48^. In brief, buffy coat (equivalent to 500 ml of whole blood for each) purchased from a blood bank (Taipei, Taiwan) with an approval of the Institutional Review Board was mixed with Ficoll-Hypaque and, after centrifugation, the layer of peripheral blood mononuclear cells was collected and the cells were incubated with anti-CD14 microbeads at 4–8 °C for 15 min. After washing, CD14^+^ cells were isolated using a magnetic-activated cell sorting cell isolation column (Miltenyi Biotec, Bergisch Gladbach, Germany). The purified monocytes were cultured in medium containing 800 U/ml GM-CSF and 500 U/ml IL-4 at a cell density of 1 × 10^6 ^cells/ml. The culture medium was changed every other day with fresh medium containing GM-CSF and IL-4, and cells with a purity greater than 92% after 5–7 days of culture were used in the experiments^48^. Human lung epithelial cell line A549 (Bioresource Collection and Research Center, Taiwan) was cultured in F12 medium (Gibco-BRL, Life Technologies Corporation, Carlsbad, CA, USA) containing 10% fetal bovine serum (FBS, Gibco-BRL) in a humidified atmosphere containing 5% CO_2_ at 37 °C.

### DENV preparation and infection

Preparation of DENV has been previously described^48^. In brief, DENV2 strains New Guinea C, 16681 and wild-type local Taiwanese strain PL046, and DENV1, DENV3 and DENV4 subtypes, were propagated in C6/36 mosquito cells in RPMI containing 5% heat-inactivated FBS and maintained at 28 °C for 7 days. Preparation of mock-conditioned medium was done using the same procedures, except that buffered saline was substituted for virus inoculation. Virus titers in supernatants were determined by plaque-forming assays, and the viral stocks were stored at −70 °C until use^48^. Unless otherwise specified, DCs (1 × 10^6^/mL in culture medium) were infected with mock or DENV at a MOI of 5 for 2 h at 37 °C. After viral adsorption, cells were washed and cultured with culture medium in the presence of exogenously added cytokines.

### Determination of virus titer

Determination of virus titer was done according to methods described in our previous report^17^. Various dilutions of virus were added to 80% confluent baby hamster kidney cells and incubated at 37 °C for 2 h. After adsorption, the cells were overlaid with 3 ml of RPMI 1640 containing 1% low-melting-temperature agarose (SeaPlaque; FMC BioProducts, Philadelphia, PA, USA), 1% penicillin, 1% streptomycin and 2% FBS. The cells were incubated for 7 days, fixed with 2% formaldehyde and stained with 0.5% crystal violet. The numbers of plaques were counted, and the results were recorded as plaque-forming units per milliliter.

### Quantitative RT/PCR (qRT/PCR)

Total RNA from treated cells was isolated with TRIzol reagent (Invitrogen, Carlsbad, CA, USA), as described in our previous report^18^. RNA concentrations were measured using Nanodrop (ND 1000 V.3.1.0). Reverse transcription of purified RNA was performed using a random primer (Applied Biosystems, Life Technologies Corporation, Carlsbad, CA, USA). The cDNA was prepared for further analysis with quantitative real-time PCR, with the aid of fluorescent LightCycler 480 SYBR Green I Master mix (Roche Diagnostics Corp., IN, USA), and analyzed with the LightCycler 480 System (Roche). All values were normalized to the level of GAPDH mRNA. All assays were performed in triplicate and repeated in three independent experiments. The primers used are shown in Supplementary Table.

### Flow cytometry

The method for determining expression of CD80, CD86, CD83, HLA-DR and CCR7 has been previously described^22^,^48^. Human DCs were collected and washed twice with cold PBS and then stained with phycoerythrin-conjugated mAbs to CD80 or HLA-DR or fluorescein isothiocyanate-conjugated mAbs recognizing CD86 or CD83 at 4 °C for 1 h. The cells were then analyzed and quantified using flow cytometry. The isotype-matched controls were purified mouse IgG1 (BD Pharmingen). For determination of CCR7 expression, cells were washed twice with cold PBS and incubated with CCR7 antibody at 4 °C for 30 min. After washing, biotin-attached anti-mouse IgG/IgM antibodies were added and incubated for another 30 min. Finally, cells were washed twice with cold PBS and stained with streptavidin PE at 4 °C for 30 min for flow cytometry analysis. Data were processed and analyzed with CellQuest software (BD Biosciences).

### Nuclear extract preparation

Nuclear extracts were prepared as previously described^48^. Briefly, the treated cells were left at 4 °C in 1 ml of buffer A (10 mM HEPES, pH 7.9, 10 mM KCl, 1.5 mM MgCl_2_, 1 mM DTT, 1 mM PMSF and 3.3 μg/ml aprotinin) for 1 h, with occasional gentle vortexing. Swollen cells were centrifuged at 14,000 rpm for 3 min. After removal of the supernatants (cytoplasmic extracts), the pelleted nuclei were washed with 300 μl buffer A, and cell pellets were then resuspended in 30 μl buffer C (20 mM HEPES, pH 7.9, 420 mM NaCl, 1.5 mM MgCl_2_, 0.2 mM EDTA, 25% glycerol, 1 mM DTT, 0.5 mM PMSF and 3.3 μg/ml aprotinin) and incubated at 4 °C for 2 h, with occasional vigorous vortexing. The mixtures were centrifuged at 14,000 rpm for 20 min, and the supernatants were used as nuclear extracts.

### EMSA

EMSA was performed as previously described^48^. Oligonucleotides containing NF-κB, AP-1 and the SP-1 binding site were purchased and used as DNA probes (Promega, Madison, WI, USA). The probes were radiolabeled with [γ-^32^p]ATP by using T4 kinase (Promega). For the binding reaction, the radiolabeled probe was incubated with 5 μg of nuclear extracts. The binding buffer contained 10 mM Tris-HCl (pH 7.5), 50 mM NaCl, 0.5 mM EDTA, 1 mM DTT, 1 mM MgCl_2_, 4% glycerol and 2 μg poly(dI-dC). The binding reaction proceeded for 20 min at room temperature before radiolabeled probes were added. Whenever competition assays were performed, a 100-fold molar excess of non-radiolabeled, competitive oligonucleotides (wild-type or mutant) were added and were pre-incubated with nuclear extracts for 30 min before adding radiolabeled probes.

### Western blotting

Enhanced chemiluminescence Western blotting (Amersham, GE Healthcare Life Science, Uppsala, Sweden) was performed as previously described. Briefly, equal amounts of proteins were analyzed on 10% SDS-PAGE and transferred to a nitrocellulose filter. For immunoblotting, the nitrocellulose filter was incubated with TBS-T containing 5% nonfat milk for 1 h and then blotted with antisera against individual proteins overnight. After washing with TBS-T, the filter was incubated with secondary Ab conjugated to horseradish peroxidase for 1 h. The filter was then incubated with the substrate and exposed to an X-ray film. After scanning, the intensity of bands on Western blots or EMSA were compared by using Alpha DigiDoc 1201 software.

### Chemotaxis assay

Chemotaxis assays were performed according to our previous report^23^. In brief, DCs treated under the different conditions migrated through a polycarbonate filter (pore size 5 μm) in 24-well transwell chambers (Corning Costar, Corning Incorporated Life Sciences, Tewksbury, MA, USA). The lower chamber of the transwell cassette contained serum-free RPMI containing 600 μl of 0.1% BSA with or without 100 ng/ml CCL19 or CCL21 (R&D Systems). DCs (1 × 10^5^) in 100 μl of serum-free medium containing 0.1% BSA were loaded in the upper chamber and incubated for 3–5 h at 37 °C. Then, cells migrating from the upper chamber to the lower chamber were counted by flow cytometry. CellQuest software was used to determine the acquired events during a fixed time period of 60 s in a FACScan.

### Knockdown of IFN-λR1, TLR-3, TLR-7, TLR-8 and IRF-3 by siRNA silencing

For knockdown experiments using A549 cells, all siRNAs (Stealth RNAi siRNA, Invitrogen, Life Technologies Corporation, Carlsbad, CA, USA) were transfected using RNAiMAX (Invitrogen) according to the manufacturer’s instructions. After cell growth reached about 50% confluence in antibiotic-free F12 medium, the cells were transfected with 10 nM siRNA. For knockdown of IFN-λR1, TLR-3, TLR-7, TLR-8 or IRF-3 in DCs, all siRNA duplexes were transfected by electroporation, using a BTX cuvette (Harvard Apparatus, Inc., Holliston, MA, USA) according to the manufacturer’s instructions. Cells were collected and resuspended in modified Eagle’s Minimum Essential Medium (Opti-MEM, Invitrogen, Life Technologies Corporation, Carlsbad, CA, USA) containing 300 nM siRNA. After transfer to the cuvette, the cells were electroporated with one pulse at 300 V for 3 ms. To determine the efficiency of protein knockdown, at 48 h post-transfection, cells were lysed in RIPA buffer and immunoblotted with the indicated Abs. The siRNA duplexes sequences used are shown in Supplementary Table.

### Determination of cytokines and chemokines by ELISA

Standard ELISA methods were used to measure concentrations of IFN-λ1 (R&D Systems), and other cytokines and chemokines, including IL-12p40, MIP-1α, MIP-1β, MCP-2, IL-6, IL-8, IL-10, RANTES and IL-1β (R&D Systems).

### Constructs of pCR3.1-NS-flag and their overexpression in A549 cells

The plasmids pCR3.1-NS1-flag, pCR3.1-NS2A-flag, pCR3.1-NS2B-flag, pCR3.1-NS3-flag, pCR3.1-NS4A-flag, pCR3.1-NS4B-flag and pCR3.1-NS5-flag were gifts from Dr. Lin Yi-Ling (Institute of Biomedical Sciences, Academia Sinica, Taiwan). The plasmids were transfected into A549 cells (1 or 2 × 10^5^/ml) by use of X-tremeGENE HP DNA Transfection Reagent (Roche, Penzberg, Upper Bavaria, Germany). DNA plasmids were mixed with the provided reagent at a ratio of 1:4 in Opti-MEM (Invitrogen). After incubation for 15–30 min at room temperature, for complex formation, the mixture was added drop-wise to the cells.

### Statistical analysis

The results were expressed as the mean ± SD of triplicate experiments. Statistical comparisons were performed by using Student’s t test or one-way analysis of variance (ANOVA). When ANOVA showed significant differences between groups, Bonferroni’s post-hoc test was used to determine the specific pairs of groups that significantly differed. A p value of <0.05 was considered to indicate statistical significance. Asterisks indicate values that are significantly different from control (*p < 0.05; **p < 0.01; ***p < 0.001, ****p < 0.0001).

## Additional Information

**How to cite this article**: Hsu, Y.-L. *et al*. Dengue virus infection induces interferon-lambda1 to facilitate cell migration. *Sci. Rep.* **6**, 24530; doi: 10.1038/srep24530 (2016).

## Supplementary Material

Supplementary Information
