# Supplementary material for: Dengue virus infection induces interferon-lambda1 to facilitate cell migration
Source: Sci Rep. 2016 Jul 26;6:24530. doi: 10.1038/srep24530 (PMC4960520; doi:10.1038/srep24530)
Supplement: Supplementary Information [file srep24530-s1.doc]

**Dengue virus infection induces interferon-lambda1 to facilitate cell migration**

Yu-Lin Hsu ^a^, Mei-Yi Wang ^b^, Ling-Jun Ho ^c^, and Jenn-Haung Lai ^b, d, *^

^a^ Institute of Preventive Medicine, National Defense Medical Center, Taipei, Taiwan, R.O.C.

^b^ Division of Allergy, Immunology, and Rheumatology, Department of Internal Medicine, Chang Gung Memorial Hospital, Chang Gung University, Tao-Yuan, Taiwan, R.O.C.

^c^ Institute of Cellular and System Medicine, National Health Research Institute, Zhunan, Taiwan, R.O.C.

^d^ Graduate Institute of Clinical Research, National Defense Medical Center, Taipei, Taiwan, R.O.C.

^*^ **Correspondence address**:

Jenn-Haung Lai, MD, PhD, Division of Allergy, Immunology, and Rheumatology, Department of Internal Medicine, Chang Gung Memorial Hospital, Chang Gung University, Tao-Yuan, Taiwan, R.O.C.

**Supplementary Figures**

**Supplementary Figure 1**

**
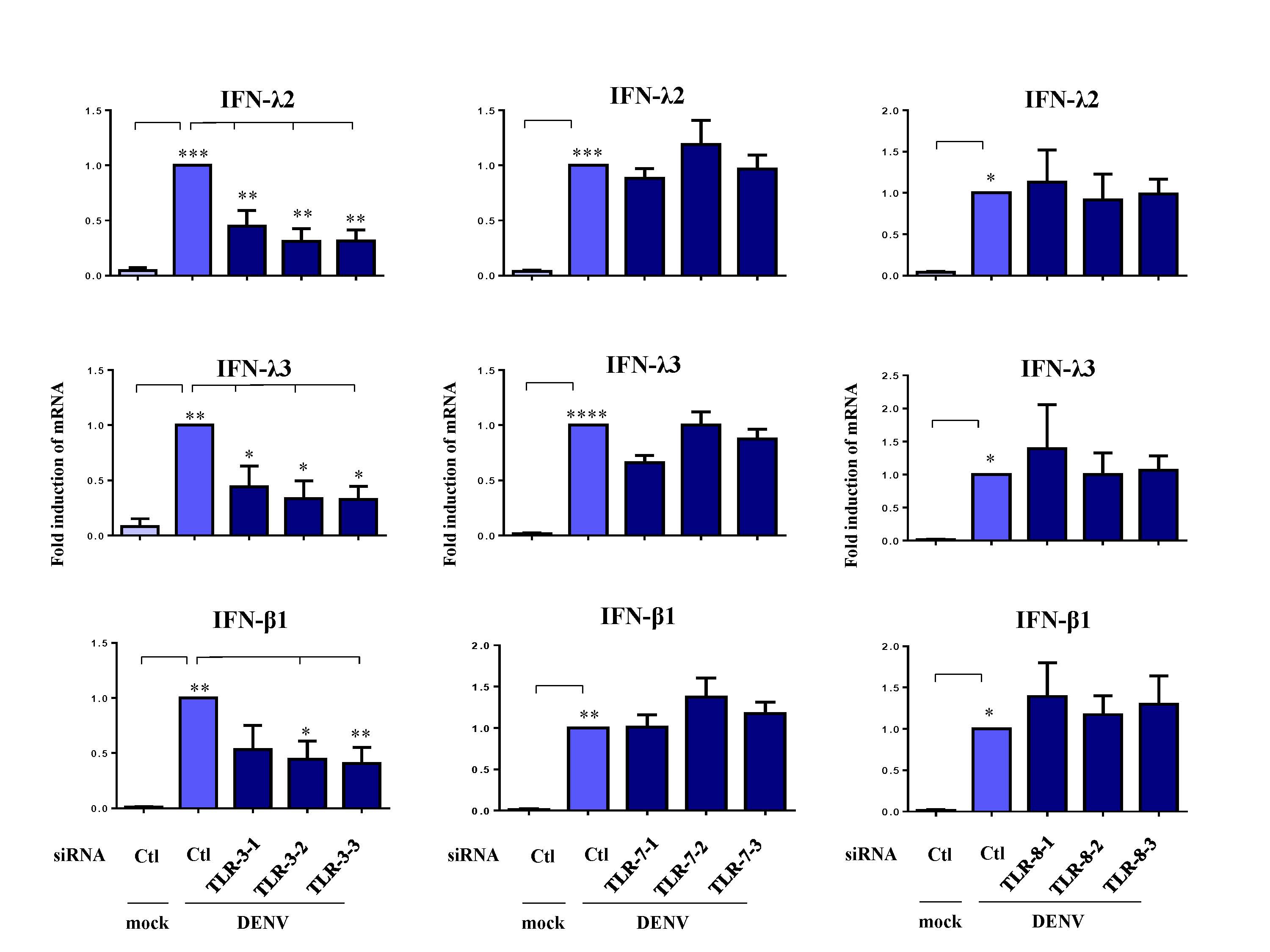
**

**Supplementary Figure 1.** Effects of IFN production in knockdown of TLR-3, -7 or -8 in DCs. Human DCs were transfected with control siRNA (siCtl) or three sets of duplexes of TLR-3, -7 or -8 siRNA for 24 h and then infected by mock or DENV for an additional 48 h. Cells were collected and the levels of IFN-λ2, IFN-λ3 and IFN-β1 mRNAs were determined by quantitative RT/PCR. The data show results pooled from at least three independent experiments. *p < 0.05, **p < 0.01, ***p < 0.001, ****p < 0.0001.

**Supplementary Figure 2**

**
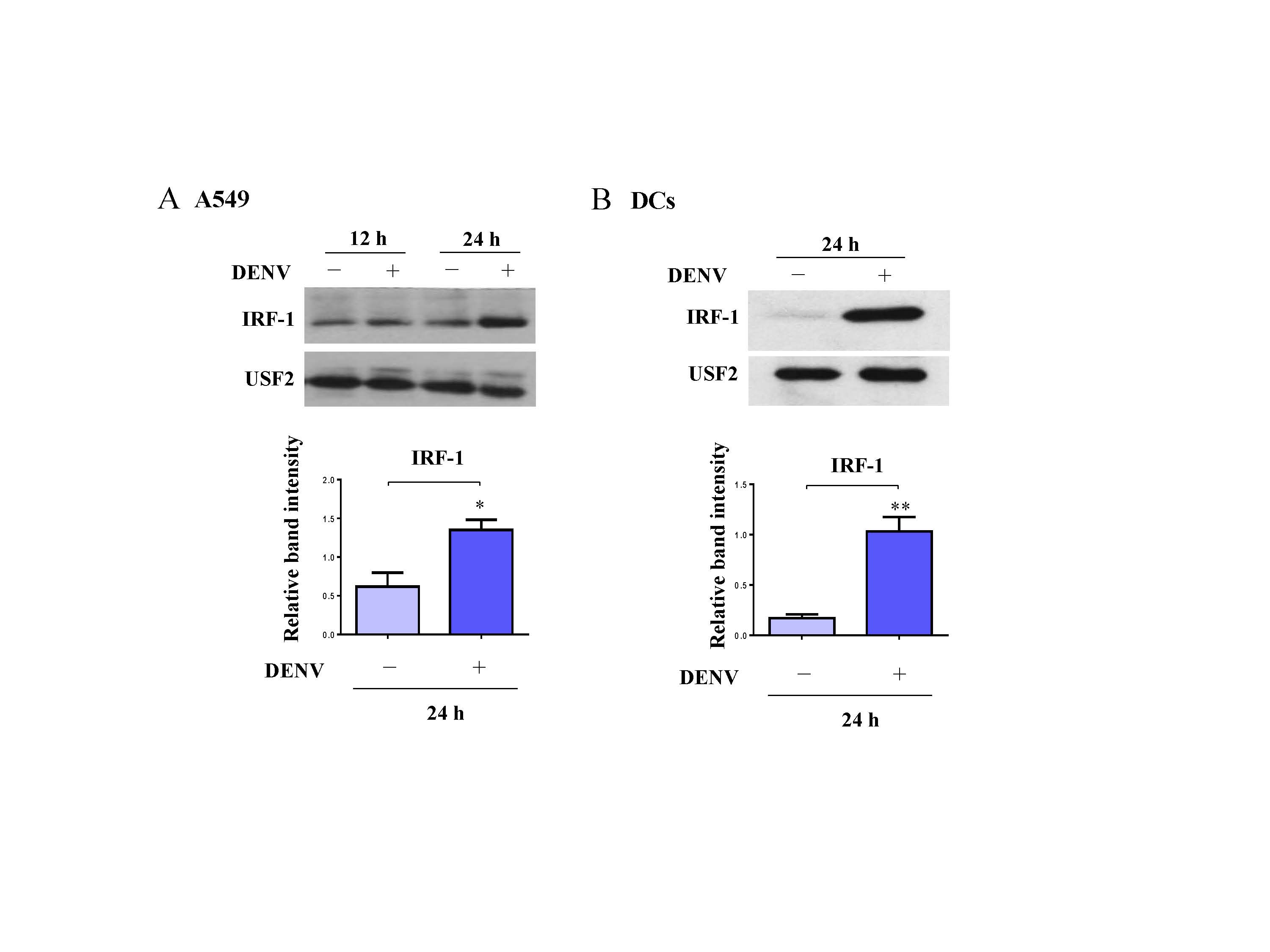
**

**Supplementary Figure 2.** Enhanced nuclear translocation of IRF-1 in both A549 cells and DCs following DENV infection. The A549 cells (A) or human DCs (B) were infected by mock or DENV for different periods of time and the nuclear extracts were prepared for the determination of IRF-1 or USF2 levels by Western blotting. The data show results pooled from at least three independent experiments. *p < 0.05, **p < 0.01.

**Supplementary Figure 3**

**
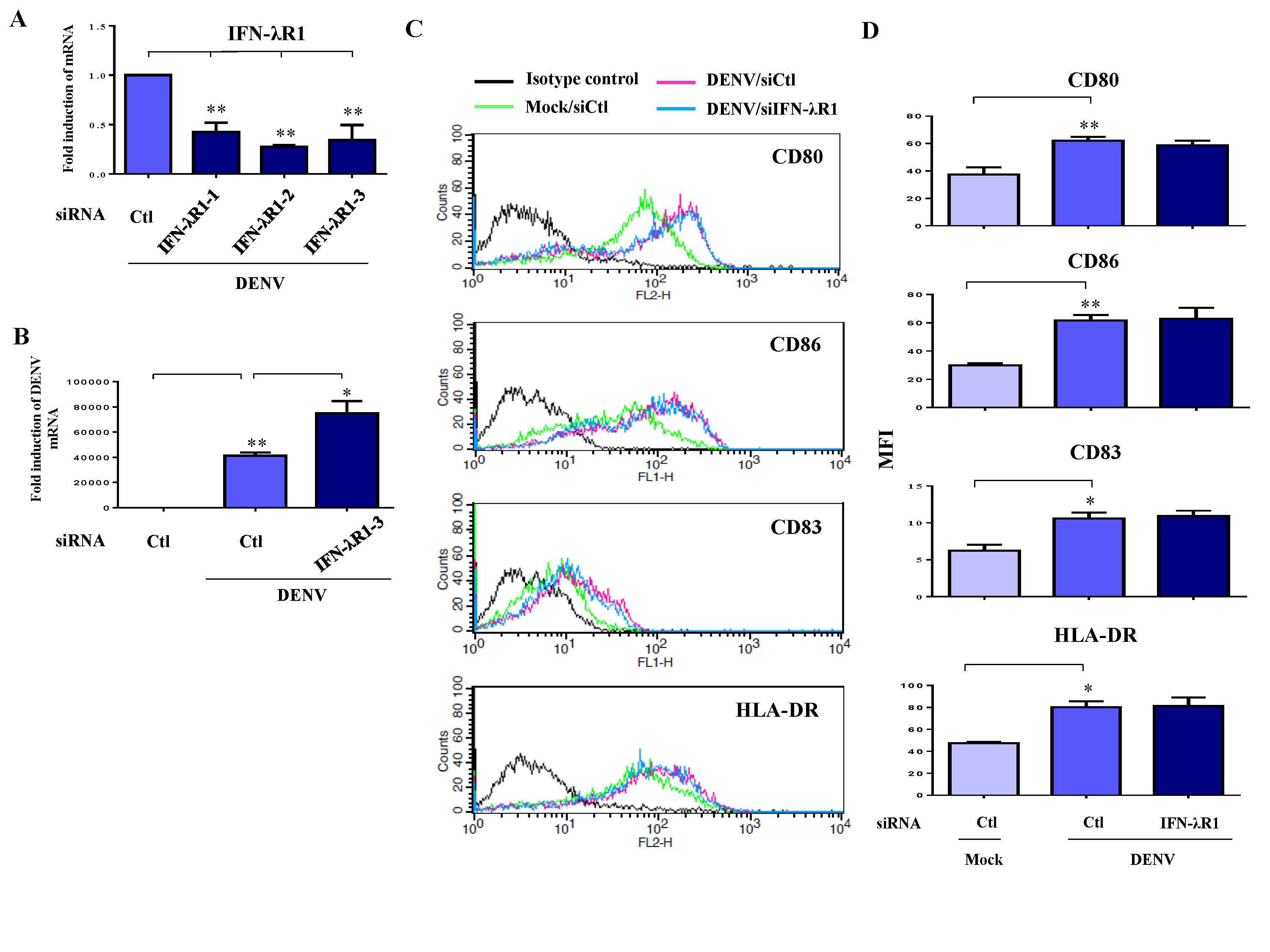
**

**Supplementary Figure 3.** IFN-λR1 knockdown increased viral mRNA level and did not affect expression of maturation or activation markers on DENV-infected DCs. Human DCs were transfected with control siRNA (siCtl) or three sets of duplexes of IFN-λR1 siRNA for 24 h and then infected with DENV for an additional 48 h. Cells were collected, and levels of IFN-λR1 mRNA were determined (A). The expression of viral mRNA infected by mock or DENV in the presence or absence of knockdown of IFN-λR1 was determined (B). Human DCs were transfected with control siRNA (siCtl) or IFN-λR1 siRNA (siIFN-λR1-3) for 24 h and then infected by mock or DENV for an additional 48 h. Cells were collected for measurement of expressions of CD80, CD86, CD83 and HLA-DR by flow cytometry (C). The results pooled from at least three independent experiments are shown (D). The analysis was performed by ANOVA, as described in the Methods. *p < 0.05, **p < 0.01. Ctl, control; MFI, mean fluorescence intensity.

**Supplementary Figure 4**

**
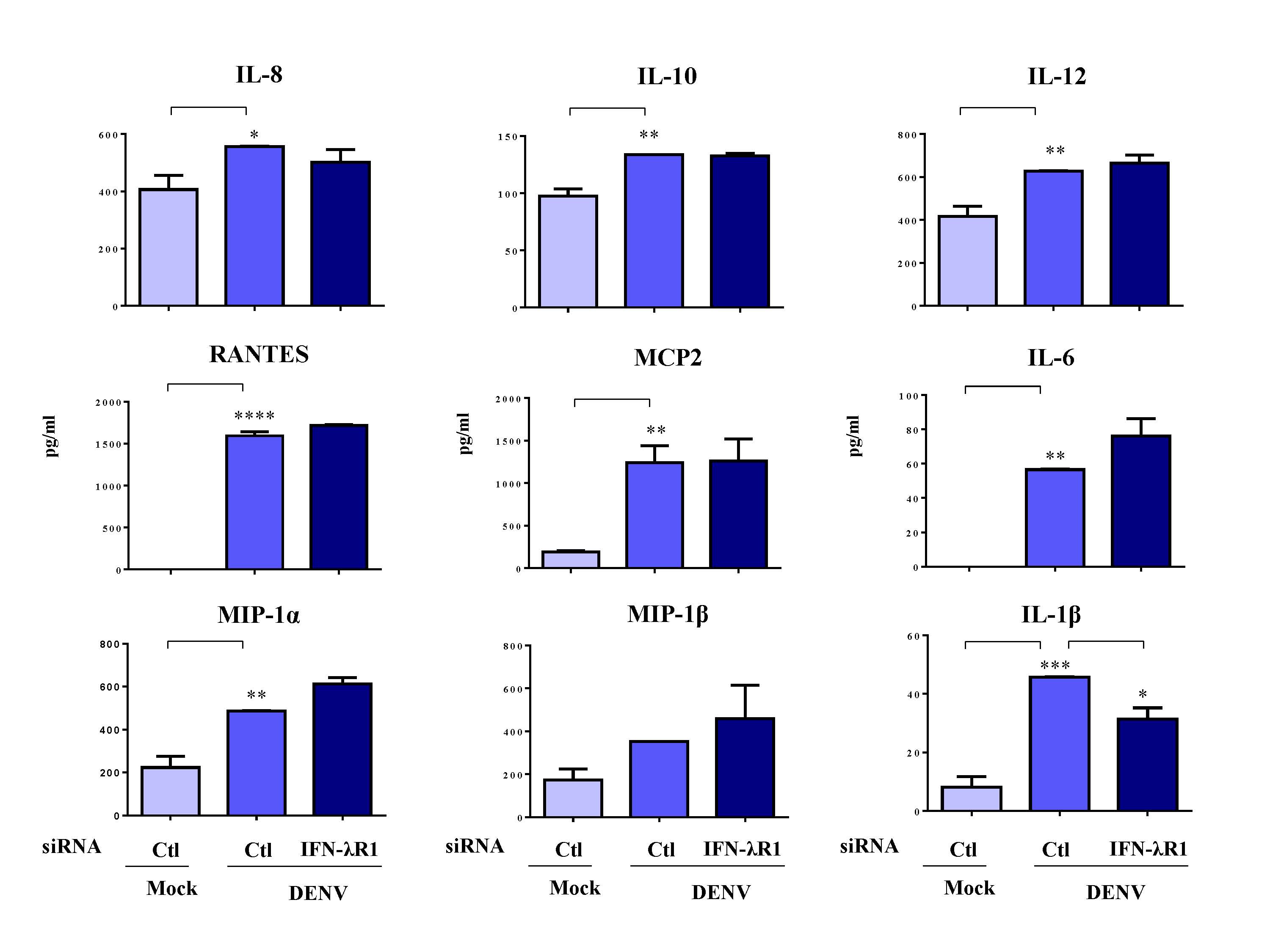
**

**Supplementary Figure 4.** Production of cytokines in IFN-λR1–deficient cells infected by DENV was measured. Human DCs were transfected with control siRNA (si-Ctl) or IFN-λR1 siRNA for 24 h and then infected with DENV for an additional 48 h. Supernatants were collected and the cytokine levels were determined by ELISA. The results pooled from at least three independent experiments are shown. *p < 0.05, **p < 0.01, ***p < 0.001, ****p < 0.0001. Ctl, control.

**Supplementary Table**

**Supplementary Table. Primers and siRNA duplex** sequences for quantitative RT/PCR or gene knockdown

| Primer sequences of genes for quantitative RT/PCR | | | | | | |
| --- | --- | --- | --- | --- | --- | --- |
| Gene | Primer sequence | | Gene | | Primer sequence | |
| IFN-λ1 | sense 5’-GAGGCCCCCAAAAAGGAGTC-3’  antisense 5’-AGGTTCCCATCGGCCACATA-3’ | | TLR-7 | | sense 5’-CTTGGCACCTCTCATGCTCT-3’  antisense 5’-GTCTGTGCAGTCCACGATCA-3’ | |
| IFN-λ2 | sense 5’-AATTGTGTTGCCAGTGGGGA-3’  antisense 5’-GCGACTGGGTGGCAATAAAT-3’ | | TLR-8 | | sense 5’-AGTTTCTCTTCTCGGCCACC-3’  antisense 5’-GGAACATGTTTTCCATGTTTCTGT-3’ | |
| IFN-λ3 | sense 5’-CCCTGGGGGATGTCTTGGA-3’  antisense 5’-GCACAACCTTGACCATCACG-3’ | | IRF-3 | | sense 5’-TGGGCCCCCAGATCTGATTA-3’  antisense 5’-TGGGCCCCCAGATCTGATTA-3’ | |
| IFN-β1 | sense 5’-CGCCGCATTGACCATCTA-3’  antisense 5’-GACATTAGCCAGGAGGTTCT-3’ | | DENV2 | | sense 5’-CTCTCAGTGAACTGCCGGAGACC-3’  antisense 5’-CGTACCATAGGAGGATGCTAGCCG-3’ | |
| IFN-λR1 | sense 5’-CCAGCCAGTCCAGATCACTCT-3’  antisense 5’-ACAGCAGTATCAGAAGCGATGG-3’ | | CCR7 | | sense 5’-GGACCTGGGGAAACCAAT -3’  antisense 5’-GCCAGGTTGAGCAGGTAGGT-3’ | |
| TLR-3 | sense 5’-TTGCCTTGTATCTACTTTTGGGG-3’  antisense 5’-TCAACACTGTTATGTTTGTGGGT-3’ | | GAPDH | | sense 5’-AGGTGAAGGTCGGAGTCAAC-3’  antisense5’-CCATGTAGTTGAGGTCAATGAAGG-3’ | |
| siRNA duplex sequences for gene knockdown | | | | | | |
| Gene | | siRNA sequence | | Gene | | siRNA sequence |
| IFN-λR1-1 | | CAGCCCUACAUUGAACCACCUUCUU | | TLR-7-3 | | GAAGCCUCAAGAAUCUGAUUCUUAA |
| IFN-λR1-2 | | CAGACACUGACCUUCUGCUGGGAAA | | TLR-8-1 | | GCCUCUGUUACUGACUGGGUGAUAA |
| IFN-λR1-3 | | GGGUGGAGUCCGAAUACCUGGAUUA | | TLR-8-2 | | GCCAGAGUCUUUGACAGAACUUAGU |
| TLR-3-1 | | GCAAACCCUGGUGGUCCCAUUUAUU | | TLR-8-3 | | GGUAUACAAUCAAAUGGCUUGAAUA |
| TLR-3-2 | | CCUGAGCUGUCAAGCCACUACCUUU | | IRF-3-1 | | AACCUUGACCAUCACGAGCCUCUUG |
| TLR-3-3 | | CCACCACCAGCAAUACAACUUUCUU | | IRF-3-2 | | CAAGGACCCUCACGACCCACAUAAA |
| TLR-7-1 | | GGACACUGAAGAGACAAAUUCUUAU | | IRF-3-3 | | CCCAGGAAGACAUUCUGGAUGAGUU |
| TLR-7-2 | | GGCUUCUUUCAUGUCUGUUAAUGAA | |  | |  |
